# Online sales adoption and financial resilience in Sub-Sahara Africa: the moderating role of ownership and enterprise size during Covid-19 crisis

**DOI:** 10.1186/s43093-022-00154-4

**Published:** 2022-09-30

**Authors:** Joseph Ato Forson, Samuel Gameli Gadzo, Emmanuel Atta Anaman, Abass Adams

**Affiliations:** Department of Applied Finance and Policy Management, University of Education, Winneba, P.O. Box 25, Winneba, Ghana

**Keywords:** Covid-19 pandemic, Enterprise size, Financial resilience, Online sales, Ownership type, Digital economy, H12, L25, O33

## Abstract

It is an established fact that the Covid-19 pandemic had a persistent economic uncertainty effect than health uncertainty. In this study, the researchers examined the effects of expanding sales online on the financial resilience of enterprises in sub-Sahara Africa (SSA) during the economic downturn. The researchers measured financial resilience by the extent of sales and cash-flows decline during the pandemic. The researchers collected 4751 unweighted data from the World Bank’s Enterprise Survey and it Covid-19 follow-up survey. Findings from the bivariate probit model and the predictive margin probabilities showed that most enterprises in SSA adopted or expanded proportion of sales online during the pandemic. Increasing the proportion of online sales exerted a decreasing effect on sales and cash-flow declines and thus improved financial resilience at a threshold of 40% during the pandemic. Large enterprises were observed to be more resilient than small and medium enterprises, yet domestic and foreign enterprises had the same level of financial resilience during the pandemic. For enterprises in Africa to realise the 40% threshold of online sales, the researchers encourage enterprises to invest in advertisement for product legitimacy.

## Introduction

An economic shock, such as the Covid-19 pandemic, distorts business environment and creates a new norm for business operations. It is an established fact that the covid-19 pandemic had a persistent economic uncertainty effect than health uncertainty  since the behaviour of the virus was understood faster than the behaviour of economic entities during the crisis [1]. Lorettu et al. [18] referred to the Covid-19 pandemic uncertainties as a *perfect storm* to both health and economic entities. The level of economic or business uncertainty was very high at the early stages of the pandemic since rational expectations were hard to form, and the pandemic containment measures were direct affront on economic activities of all forms [18]. This level of economic uncertainty could be expected in any future pandemic or crisis of the Covid-19 characteristics, hence the need to understand the behaviour of enterprises and households at the early stages of the pandemic. From the enterprises’ perspective, copping strategies are aimed at either sustaining production, given that demand is assured, or boosting sales, given that production is assured. One such coping strategy was digitalisation that allowed both enterprises and their customers to trade online.

Adopting sales online was supposed to have its own positive and negative consequences. On the positive side, enterprises that sell more of their products online have access to unlimited customer base on which Covid-19 containment measures may impact less [11]. Hence, the change in the geographical distribution of customers owed to the containment measures such as lockdowns and temporal closure of business may have less impact on demand. Established online sales routes also offer reliable sales avenue that could reduce fluctuations in sales as compared to direct sales due to the effects of Covid-19 containment measures [11]. Online sales, therefore, could impact positively on the financial performance or resilience of an enterprise in the areas of sales and cash-flow sustenance. With regard to cash-flow difficulties, online sales may allow enterprises to determine potential demands ahead of time and in some cases receive advance payments that reduce the cash conversion cycle (CCC) and minimise liquidity challenges. Thus, demand forecast become more reliable as more sales are done online than in-person for which the enterprise may have no clue whatsoever about when sale will occur and where it will occur.

The contribution of this paper therefore is to examine the effects of expanding sales online on the financial resilience of enterprises in sub-Sahara Africa (SSA) during the economic downturn. The researchers measured financial resilience by the extent of sales and cash-flow decline during the Covid-19 pandemic. Our decision to undertake such a study is borne out of the fact that, sub-Sahara Africa is plagued with some challenges with regard to selling products online and this could worsen the plight of existing enterprise especially in times of shocks such as the recent pandemic. These challenges could be presented in twofold. First, going online as a coping strategy to a crisis situation could require high initial set-up cost or may require that such services are outsourced [29]. For the case of initial set-up cost, the *winner’s curse phenomenon* could imply where the cost of getting the product sold online may be higher than selling it onsite or in person; and hence the benefits of selling more online may be eroded. Also, outsourcing could come with additional service charges that consumers may implicitly consider as part of the cost of the product. The perceived high transaction cost from online activities could affect the volume of demand as compared to other closed substitute products sold in brick-and-mortar or offline sales. Second, the decision of an enterprise to go online must coincide with the decision of the customer-based, who may be the direct patrons of the internet for purchasing activities [30]. If the customer-based slag in their digitalisation process, then the initial set-up cost of enterprises may not generate enough sales to offset the cost and be able to generate profit [30]. The resolution to such impasse about how online sales could impact financial performance therefore becomes an empirical issue that require evaluation in studies such as the current study.

The paper is organised as follows: section one is the background of the study that sets out the motivation for undertaking the study. The contribution the paper seeks to make as against the implications for not understudying this phenomenon is outlined in this section. “Related literature review and hypotheses development” section reviews the literature on the resource-based view and transaction cost theory. Further attempt is made to draw on the empirical relation that borders on online sales (e-commerce) during the pandemic period. Consequentially, two hypotheses are developed from the empirical reviews on online sales and financial resilience on one side, and the mediating and moderating role of enterprise size and ownership on online sales performance on another side. The research method and design is presented in “Research method and design” section. The data source and model specification are jointly presented in this section together with the descriptive statistics. The researchers present and discuss the research findings in “Results and discussions” section. The moderating role of enterprise size and ownership type of online sale on financial resilience is captured as part of the discussion in this section. The paper concludes in “Conclusions and recommendations” section with some policy recommendations.

## Related literature review and hypotheses development

### Theoretical perspective

Theoretically, the study was grounded in the resource-based view of enterprise performance and transaction cost theory. Resource-based view stressed on the firm’s internal resources and capabilities in relation to its external environment [22]. The resource-based view is concerned with how enterprises used their capabilities and internal resources to create a niche or competitive advantages so as to boost performance [22]. During the pandemic, enterprises had to leverage on the internal resource which include information communication technology (ICT) that they developed prior to the pandemic or during the pandemic [4]. Chaffy [3] stated that leveraging on ICT resources to sell more online could create both tangible and intangible benefits, with the tangible ones including increase in sales and reduction of administrative, marketing and supply chain costs. Ciarniene and Stankeviciute [4] concluded that the combination of both tangible and intangible benefit of increasing competitiveness through online sales may result in improved financial performance or profitability.

Yakup et al*.* [32] summarised the benefit of online sales to include providing products and services conveniently, pricing, the ability to target diverse demographics at once, and allowing customers to easily research products and services to expedite purchasing decision. All the areas identified by Yakup et al*.* [32] became very necessary during the pandemic, especially as the geographical distribution of population were significantly altered.

The effects of increasing online sales on financial performance may as well depend on its relationship with transaction cost. That is, whether selling more online improve financial resilience or not depends on how it affects the transaction cost of sales as well as its effect on the cash conversion cycle. The main focus of the current study was to examine and explain how the proportion of online sales influences financial resilience of enterprises during the deteriorated Covid-19 business environment.

### Online sale and financial resilience during pandemic

The extant literature suggests that there are several studies on online activities and electronic commerce (e-commerce) prior to the pandemic and few within the pandemic. For instance, Lockett (2018) identified online marketing strategies and online content strategies as among the strategies that SMEs can use to boost sales revenue. Toombs and Harlow [27] had earlier made a similar observation, but George et al. [9] were sceptical about the effects of online activities on the performance of SMEs. Resnick et al. [24] added that SMEs perceive online activities as costive as compared to large enterprises. That is, SMEs may not cover the sales volume threshold that will ensure internal economy of scale necessary to bring cost down for improved sales performance.

Duch-Brown et al. [5] acknowledged that online sales constitute both expansion in sales and diversion of traditional sales. For financial resilience during crisis, such as the Covid-19 pandemic and its containment measures such as social distancing and lockdowns, diversion of traditional sales to online sale was the major motivation for enterprises seeking to sell more online. Purnomo and Adiguna [23] in an attempt to explore the resilience of small- and medium-sized enterprise (SMEs) in Indonesia argued that the Covid-19 pandemic did trigger the emergence of both new constraints and opportunities. The constraint they explained had to do with the interruption in SMEs business model. Regarding the opportunities, they explained that the quest for survival, continuity and growth engendered SMEs to draw from their resourcefulness and firm-level strategies to cope with the constraints. The coping strategies may include leveraging on the digital economy through social media and other online platforms to enhance sales volumes.

Women enterprises were not left-off the hook of the devastation caused by the pandemic. In a study Anggadwita et al. [2], which sought to explore women resilience in the face of a pandemic, proposed a framework for factors necessary to enhance the resilience of women family businesses. The study stressed on the strategic decision-making of women, that may be evident in their adaptive capacity, strategy renewal and appropriate capacity as key resilience factors [6]. The study among other things concluded that women have long-term orientation towards the sustainability of family business and can overcome conflicts through adaptive mechanisms. Adapting to online platforms as a mechanism to enhance sales volumes thus becomes inevitable [10]. We hypothesise the following based on the extant literature on the connection between online sales and resilience during pandemic:

*H1*: strategies to increase online sales during pandemic is associated with high sales performance and guaranteed financial resilience of enterprises

### Moderating and mediating effects of enterprise ownership and size on online sales performance

According to Jovanovic et al. [15], there is a positive mediating effect of online sales channel on the effects of online sales on performance. Inyang and Jaramillo [14] examined the performance implication of sales strategy implementation by salesperson with data from a sample of 190 business to business salespersons in different industries. They developed hypotheses on this relationship and concluded that different types of salesforce may impact on salesperson market and technical knowledge differently or in a contrasting manner. They therefore asserted that when salesperson implement sales strategy as part of the sales process, it has positive effects on their sales performance. Moen et al. [19] examined the role played by product category as a moderator in online reviews and sales enhancement using natural language processing (NPL). They found reviews to significantly impact on sales performance, but were quick to add that such effects were plausible through the interaction with product category and its popularity (see [12, 16, 25].

Wentrup [31] argued that finding a suitable balance between an online and offline presence is critical to the success of internationalisation efforts. The result of Wentrup [31] implies that foreign owned enterprises could have improved performance by adopting online sales and activities. The outcome of the current study expands to debate to imply that the advantages of complementing offline sales with online sales could equally be reaped by domestic enterprises irrespective of size. The observation that innovation into online sales could improve the performance of SMEs are well documented [7, 8, 26]. Moreno et al. [20] earlier observed in Belgium that online activities are predominant in large enterprises as compared to SMEs and accordingly observed that online activities have limited impact on the performance of SMEs but significantly influence the performance of large enterprises positively (see Peng et al., [21]). Based on these studies, we hypothesised that:

*H2*: an increase in online sales through products popularity is associated with sales performance regardless of enterprise ownership and size.

## Research method and design

### Data and model specification

The study was purely quantitative following the positivist research philosophy. The design was explanatory and cross-sectional in time dimension. The dataset from the World Bank Covid-19 Follow-up Survey that traced enterprises that were involved in the Enterprise Survey (WBES) prior to the pandemic was used for the analysis. The follow-up survey included eight sub-Saharan African countries (Chad [153], Guinea [150], Mozambique [601], Niger [151], Togo [150], Somalia [1144], Zambia [1202] and Zimbabwe [1200]). Somalia, Zambia and Zimbabwe had completed second wave of the survey.

Together the dataset contained 4751 unweighted observations on SSA countries; which translates to 214,695 weighted sample size. A bivariate probit (bi-probit) model was fitted as presented in Eqs. (1) and (2):

1 $${\text{Sales}}_{i} = \alpha + \gamma_{1} {\text{saleonline}}_{i} + \gamma_{2} {\text{saleonline}}\_{\text{sq}}_{i} + \beta_{1} X_{i} + e_{i}$$

2 $${\text{Cash}}_{i} = \alpha + \gamma_{1} {\text{saleonline}}_{i} + \gamma_{2} {\text{saleonline}}\_{\text{sq}}_{i} + \beta_{i} X_{i} + \varepsilon_{i}$$

where *α* is the intercept, $\gamma_{1}$ is the marginal effects of the linear term of online sales, and $\gamma_{2}$ is the marginal effects of the quadratic terms of online sales. Also, the $\beta$’s are the slope or marginal coefficients of the control variables such as the enterprise size, ownership structure, delivery service, export status, sector, proportion of employees under remote work and owners’ or managers’ gender compositions.

The model followed the seemingly unrelated regression equation (SURE) model which takes the possible endogeneity of sales and cash-flow decline into consideration through the correlation between the error terms (e and *ε*). That is, simultaneity biased was suspected which supports the use of simultaneous equation-based estimation. The appropriateness of the bivariate probit model was tested with the rho test. The maximum likelihood estimation (MLE) technique was used to estimate Eqs. (1) and (2) simultaneously.

### Descriptive statistics

The distribution of sales and cash-flow situations is presented in Table 1 with the chi-square test of dependency. The results suggested that the issue of sales and cash-flow decline was wide spread among enterprises in sub-Saharan region due to Covid-19 effects. That is, 110,569 enterprises, representing about 74.03% sampled enterprises, were estimated to have experienced both sales and cash-flow decline in the early stages of the pandemic and into the future. Only about 9.30% were resilient enough not to have experienced any of sales or cash-flow difficulties, with about 7.30% experiencing only sales decline and the remaining 9.74% experiencing only decline in cash-flow. Together, about 83.40% of the enterprises experienced decline in either sales or cash-flow within the sample period. The chi-square test of dependency suggested that a statistical significant dependency exists between sales and cash-flow declines at the five per cent significance level.

**Table 1:** Cross-tabulation of Sale and Cash-flow Decline in SSA. *Source:* authors construct based on WEBS follow-up survey (2022)

	Cash-flow		
Sales	No decline	Decline	Sum
No Decline	13,895	14,001	27,896
Decline	10,900	110,569	121,469
Sum	24,795	124,570	149,365

Chi-square test: Pearson chi2 (1) = 2.7e + 04, Pr = 0.000

N/B: frequency weight applied

**Fig. 1:**
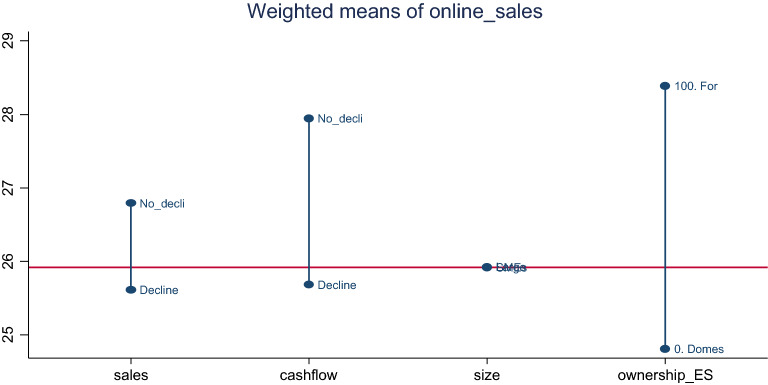
Percentage of Enterprises’ sales online based on sales, cash-flow and moderators decline in SSA. *Source*: authors’ construct based on Covid-19 ES follow-up survey (2022)

Figure 1 presents the distribution of online sales of enterprises during the Covid-19 deteriorated business environment. The results suggested that enterprises that experienced no decline in sales or cash-flow were the heavy adopters of online sales as compared to those that experienced decline in sales. That is, enterprises that experienced no decline in sales sold about 26.79% of their outputs online with a relative spread of about 0.8214%, whilst those that experienced a decline in sales sold about 25.61% of their outputs online with a spread 0.8779. Similarly, enterprises that experienced no decline in cash-flow sold about 27.95% of their outputs online with a spread of about 0.8686%; whilst those that experienced decline in cash-flow sold about 25.68% of their outputs online with a spread of about 0.8516. The trend emerged that enterprises that sold more of their output online appeared less likely to have experienced decline in sales or cash-flow during the crisis.

The results further suggested that foreign enterprises were more likely to sell more of their product online during the crisis than domestic enterprises in SSA. That is, foreign enterprises sold about 28.39% with a spread of 0.8882, whilst domestic firms sold about 24.81% of their output online with a spread of about 0.8382. The results further indicated that SMEs and large enterprises sold equal percentage of their product online (SMEs: mean = 25.91526, CV = 0.8544 and large: mean = 25.92363, CV = 0.8895) in the wake of the Covid-19 pandemic.

The observation that most enterprises, irrespective of size or ownership structure, sold a significant proportion of their sales online was consistent with the earlier observation of UNCTAD [29] and Totolo & Baijal [28] among others. That is, UNCTAD [29] observed that “the pandemic has accentuated the trend towards greater adoption of social media and growth in sales through e-commerce websites” (p.7). The study of UNCTAD [29] had 11 African countries involved in the data collection and analysis and hence relatively representative of the SSA case. Totolo and Baijal [28] asserted that “[Covid-19 pandemic] has vastly accelerated digital adoption. Online sales are no longer an option, but a necessity for brick-and-mortar businesses” (p. 1).

## Results and discussion

### Regression analysis of online sales and financial resilience

Table 2 presents the bivariate probit model of sales and cash-flow decline as endogenous variables with percentage of online sales, enterprise size, ownership structure and some control variables as exogenous variables. The Wald test of rho equal zero was rejected at the 5% significant level (rho = 0: chi2(1) = 5326.03; Prob > chi2 = 0.0000), which implies the joint model was superior to fixing two separate probit or logit models.

**Table 2:** Bi-probit model of sales and cash-flow decline and their correlates. *Source*: authors’ construct based on Covid-19 ES follow-up survey (2022)

	Coef.	St.Err.	*t*-value	*p*-value	Sig.
*Sales*					
Online_sales	0.0059	0.0011	5.52	0.000	***
Onlinesales_square	− 0.0001	0.0001	− 10.03	0.000	***
No female	0				
Female present	0.234	.028	8.46	0.000	***
No delivery	0				
Delivery	− 0.225	.016	− 13.74	0.000	***
Remote work	0.002	0	4.57	0.000	***
Manufacturing	0				
Retail	− 0.338	.021	− 15.95	0.000	***
Other services	0.027	.019	1.41	0.159	
Domestic	0				
Foreign	0.015	.017	0.88	0.378	
No exports	0				
Exports	0.044	.023	1.92	0.054	**
SMEs	0				
Large	− 0.242	.019	− 12.73	0.000	***
Constant	1.111	.024	46.69	0.000	***
*Cash flow*					
online_sales	0.0114	.0011	10.26	0.000	***
Onlinesales_square	− 0.0002	0.0001	− 13.56	0.000	***
No female	0				
Female present	0.036	0.0279	1.30	0.193	
No delivery	0				
Delivery	− 0.164	0.0171	− 9.59	0.000	***
Remote work	− 0.007	0.0003	− 22.07	0.000	***
Manufacturing	0				
Retail	− 0.079	0.0218	− 3.62	0.000	***
Other services	0.403	0.0198	20.31	0.000	***
Domestic	0				
Foreign	0.026	0.0180	1.45	0.148	
No exports	0				
Exports	− 0.225	0.0223	− 10.09	0.000	***
SMEs	0				
Large	− 0.273	0.0197	− 13.90	0.000	***
Constant	1.084	0.0243	44.51	0.000	***
/athrho	1.109	0.0152	72.98	0.000	***
Mean dependent var	0.847	SD dependent var	0.360		
Number of obs	34,979	Chi-square	3044.130		
Prob > chi2	0.000	Akaike crit. (AIC)	54,058.655		

Note. ***,** and * indicates significant at 1%, 5% and 10% level respectively.

**Fig. 2:**
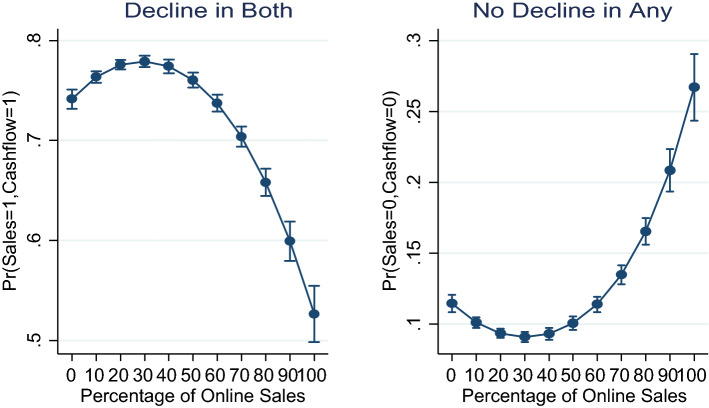
Predictive margins plot of sale and cash-flow decline. *Source*: authors’ construct based on Covid-19 ES follow-up survey (2022)

The results suggested that the percentage of enterprises’ sales online had significant direct but diminishing effects on both sales decline and cash-flow decline during the pandemic. The results implied a threshold effect at which further percentage increase in online sales begins to exert negative effects on sales and/or cash-flow decline. That is, both the sale and cash-flow models were identical in terms of shape- had inverted U-shaped. Figure 2 presents the predictive margins plot of the probability that an enterprise shall experience decline in both sales and cash-flow at respective level of online sales during the pandemic.

The plot depicts a threshold effect of about 40% of online sales before further increase in online sales could reduce both sales and cash-flow decline. The consistency of the optimal percentage online adoption was vivid in the two plots, since the probability that an enterprise that adopt online sales shall not experience either sale or cash-flow decline also increases after 40% of adoption.

**Fig. 3:**
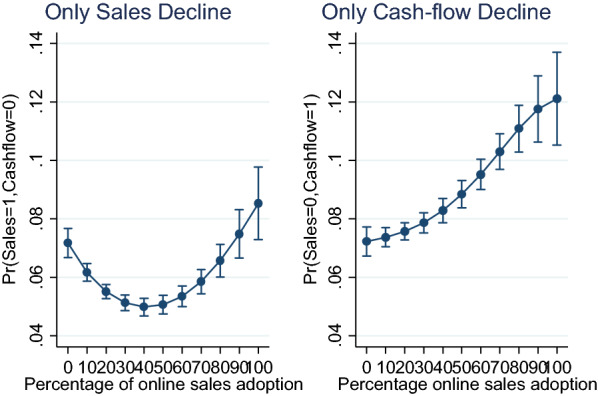
Predictive margins plot of sale and cash-flow decline in isolation. *Source*: authors’ construct based on Covid-19 ES follow-up survey (2022)

The other two possibilities were the likelihood for an enterprise to experience sales decline without experiencing cash-flow decline, and the tendency that an enterprise shall experience cash-flow decline without having experienced sales decline. These two possibilities were estimated and presented in Fig. 3.

The plot in Fig. 3 suggests that the distribution of decline in only sales is U-shaped, which implies that after about 40% of online sales adoption, an enterprise can experience sales decline without experiencing cash-flow difficulties. This could be explained by the fact that the magnitude of the decline reduces after more outputs are sold online. The case of decline in cash-flow without sales decline followed the Ogive shape, which implies that though the probability of cash-flow decline increases even online sales increase but the rate increases at a decreasing rate.

The outcome of the analysis provides support to the pre-pandemic views that increasing sales online could improve enterprises’ performance. Duch-Brown et al. [5] found that selling more online could lead to expansion in sales and diversion of traditional sales. That is, sale expands because an enterprise could reach more customers by making it product available online which expand the customer base. Also, customers that the enterprise may have lost because of change in location, as it was the case during the lockdowns of the pandemic, could still be reached through online sales. Hence sales variances could be expected to be small for enterprises with significant sales online than those that were operating mainly offline, as it was observed in the current study.

The observation that a threshold effects exist on the effects of online sales on financial resilience has been observed elsewhere in earlier studies in Canada. The DBC observed a threshold of 50% of online sales before enterprises exhibit superior performance over their peers engaged in direct sales. But the current study observed a threshold of 40% of online sales to guarantee positive effects on financial resilience of enterprises during economic downturn of the Covid-19 pandemic.

### Moderating role of enterprise size and ownership type on the effects of online sales on financial resilience

**Fig. 4:**
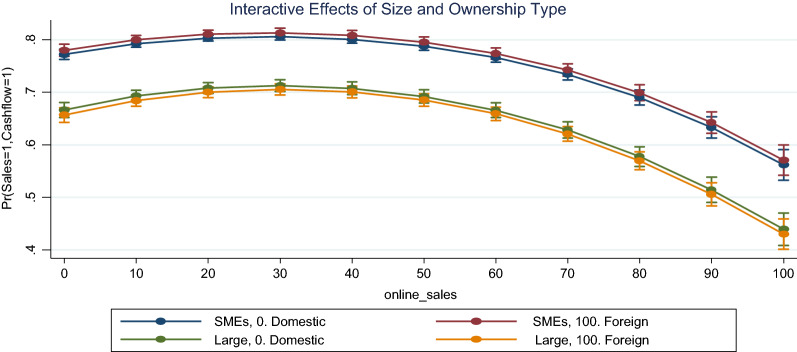
Interactive effects of size and ownership type on the relationship between online sales and financial resilience. *Source*: authors’ construct based on Covid-19 ES follow-up survey (2022)

From Table 1, the results indicated that ownership type does not significantly influence either the odds of sale decline or of cash-flow decline during the pandemic. That is, domestic and foreign owned enterprises had identical odds of experiencing sales or cash-flow decline during economic downturn in SSA. However, the results suggested that large enterprises were less likely to record sales or cash-flow decline as compared to the case of SMEs. That is, compared to SMEs the odds that a large enterprise shall experience decline in sales or cash-flow were reduced by about -0.242 and -0.273, respectively. Since ownership structure did not directly affect the financial resilience of the enterprises in SSA, it was prudent to determine whether it had indirect effects as a moderator, which was done in this study using the interactive effects of ownership and enterprise size. The results, as presented in Fig. 4, suggested that size and ownership type strongly moderate the relationship between online sales and financial resilience during the pandemic. That is, though large enterprises were more resilient than SMEs it was observed that large-foreign enterprises were relatively more resilient than large-domestic enterprises. Yet, the effects alternate in the case of SMEs where domestic SMEs were more resilient than foreign SMEs.

The fact that there are moderating and mediating effects to the relationship between online sales and enterprise performance was consistent with the outcome of the studies of Jovanovic et al., [15]. Jovanovic et al. [15] observed a positive mediating effect of online sales channel on the effects of online sales on performance. The current study observed enterprise size and ownership type as moderating the positive nonlinear relationship between proportion of online sales and financial resilience during economic shock such as the Covid-19 pandemic. Wentrup [31] argued that finding a suitable balance between an online and offline presence is critical to the success of internationalisation efforts. The result of Wentrup [31] implies that foreign owned enterprises could have improved performance by adopting online sales and activities. The outcome of the current study expands the discourse on this matter to imply that the advantages of complementing offline sales with online sales could equally be reaped by domestic enterprises irrespective of size. The observation that innovation into online sales could improve the performance of SMEs are well documented [7, 8, 26].

The outcome of the study, however, contradicts the views of Ika et al. [13] who observed that increasing online activities such as selling online have insignificant effects of enterprises financial performance. Their focus, however, was on performance in general but not resilience as was the case in the current study.

## Conclusions and recommendations

In this study, we examined the effects of expanding sales online on the financial resilience of enterprises in sub-Sahara Africa (SSA) during the economic downturn. Our analysis was purely based on quantitative technique with about 4751 unweighted data observations from the World Banks Enterprise Survey (WBES) and it Covid-19 follow-up survey. We used the bivariate probit estimation approach along with the predictive margin probabilities and plots. The outcome of the analysis suggested that a significant number of enterprises, large and SMEs and domestic or foreign, sold significant proportion of their sales online as a coping strategy to improve financial resilience during the Covid-19 pandemic. However, though the adoption rate of both large enterprises and SMEs were almost identical, the foreign enterprises adopted more online sales than the domestic enterprises. The results gave some credence to the earlier held view prior to the pandemic that foreign owned enterprises may have high absorptive capacity to take advantage of an innovative spill-over than domestically owned enterprises. That is, the opportunity to go digital became cheaper and available during the pandemic such that the major hindrance to adoption was the position of an enterprise and their ability to recognise, assimilate and adopt.

In terms of performance, the study could conclude that the proportion of online adoption initially reduces the resilience of an enterprise but improves resilience after selling more than 40% of total output online. That is, the relationship between the proportion of sales online and financial resilience during economic downturn is inverted U-shaped.

Enterprise size was observed to improve financial resilience such that large enterprises were more financially resilient than SMEs in SSA, but ownership type as foreign or domestic had no statistical significant effects on financial resilience. Enterprise size and ownership type was found to moderate the relationship between online sales and financial resilience. Comparatively, large-foreign enterprises were relatively more resilient than large-domestic enterprises but domestic SMEs were relatively more resilient.

Based on the results the study recommends that owners and managers of enterprises in sub-Sahara Africa (SSA) to increase their proportion of sales online beyond the threshold of 40% so as to reap the benefit of digital sales or e-commerce under the pandemic uncertain business environment. SMEs are encouraged to invest in infrastructure that could facilitate digital sales innovation since such innovation has promising performance effects on them just as large enterprises. Large-domestic enterprises need to reposition themselves in the digital market to reap the benefit of digitalisation just as large-foreign enterprises.

## Data Availability

Not applicable.

## Abbreviations

SSA: Sub-Sahara Africa (SSA)
SMEs: Small- and medium-scaled enterprises
WBES: World Banks Enterprise Survey
MLE: Maximum likelihood estimation
SURE: Seemingly unrelated regression equation
UNCTAD: United Nations Conference on Trade and Development
