# Correction: Online sales adoption and financial resilience in Sub-Sahara Africa: the moderating role of ownership and enterprise size during Covid-19 crisis

**DOI:** 10.1186/s43093-022-00165-1

**Published:** 2022-11-18

**Authors:** Joseph Ato Forson, Samuel Gameli Gadzo, Emmanuel Atta Anaman, Abass Adams

**Affiliations:** Department of Applied Finance and Policy Management, University of Education, Winneba, P.O. Box 25, Winneba, Ghana

## Correction: Future Business Journal (2022) 8:42 10.1186/s43093-022-00154-4

Following publication of the original article [1], the authors identified an error in the author name of Abass Adams).

The incorrect author name is: Abbas Adams.

The correct author name is: Abass Adams.

The author group has been updated above and the original article [1] has been corrected.
